# Adoption of M-Learning in Business English Course and Its Relationship to Learning Style Preferences: An Empirical Investigation

**DOI:** 10.3389/fpsyg.2022.881866

**Published:** 2022-05-04

**Authors:** Xiaojun Cao

**Affiliations:** ^1^Department of Public Courses, Xi’an Traffic Engineering Institute, Xi’an, China; ^2^Universiti Teknologi MARA (UiTM), Selangor Darul Ehsan, Malaysia

**Keywords:** inquiry learning, reflective thinking, peer communication, problem-solving skills, critical thinking skills

## Abstract

Learning around the world has been changed with the rapid development in technology which promotes the students to be more flexible and interactive with each other which has been encouraged by the mobile learning environment. Therefore, the current study intends to analyze the impact of inquiry learning, reflective thinking on problem-solving skills, and critical thinking skills with the mediation of peer communication. To carry out the study, data was collected from 378 college students in China by using survey forms. The analysis of the data and validation of the proposed hypotheses were conducted using Smart-PLS and structural equation modeling (SEM) technique. The results revealed that inquiry learning and reflective thinking affect problem-solving skills. However, inquiry learning and reflective thinking did not affect critical thinking skills. Moreover, the study found that peer communication mediated the relationship between reflective thinking, problem-solving skills, and between reflective thinking and critical thinking skills. However, peer communication did not mediate the relationship among inquiry learning as independent variable and problem-solving skills and critical thinking skills as dependent. The study has theoretically contributed by examining the impact of online learning styles on higher-order thinking skill (HOTS) in the M-learning environment. Also, the study greatly advances the literature by investigating the mediating role of peer communication. Practically, the colleges can improve the students HOTS by devising policies and educational programs focusing on learning styles.

## Introduction

With the rapid development of technology, the learning environment around the world has been changing and becoming more social, flexible, interactive, and student-oriented (Mulyadi et al., 2022). One of the learning environments that have recently gained attention is M-learning or mobile learning. Mobile learning is referred to as the learning context in which the students use their portable devices to access the mobile network to carry out their learning, and it can be either in or out of the classroom (Berweger et al., 2022). This involves the use of advanced forms of educational technologies in the physical classrooms to maximize the ability of the instructor, facilitate the learning ability of the students, and shape the ability of the students to participate in formal educational learning experiences (Müller and Mildenberger, 2021). Such experiences offered to the students are beyond the possibilities of conventional classrooms.

M-learning possesses four main features (Lu et al., 2021). First, it involves a technology-rich learning environment that gels a virtual environment with a psychical environment. Second, it provides communication and information technology tools, interaction support, and various learning and teaching activities. Third, it helps to optimize and maximize pedagogical decisions through data storage, collection, computation, and analysis of the learners. Fourth, it provides an open learning environment where learners can experience authentic learning space. Additionally, studies have found a positive influence of incorporating mobile technologies into the school curriculum on higher-order thinking skills (HOTSs; Lu et al., 2021). Moreover, the importance of HOTS has been emphasized by researchers, policymakers, educators, and the general public (Hadzhikoleva et al., 2019). Three types of HOTS have been identified by Hwang et al. (2018) as problem-solving skills and critical thinking skills.

Problem-solving is referred to as the ability to determine a problem, collect and analyze relevant information, and implement an appropriate and suitable solution (Rahman et al., 2019). Problem-solving requires multiple sources of information to come up with the most appropriate solution, and such sources are offered by M-learning with its constructive learning environments. Therefore, students encourage the use of a comfortable environment having both digital and non-digital resources (Araiza-Alba et al., 2021). Problem-solving skills acquired by the students help to achieve the academic goals and get higher aggregate in the class. Moreover, such skills not only help the students in their college life, but they can overcome and solve various problems that they may encounter ahead in their lives. Thus, one of the great advantages offered by M-learning is shaping and fostering the problem-solving skills of the students (Lane et al., 2022).

Another type of HOTS is critical thinking skills which are referred to as the ability of an individual to make a reasoned judgment based on objectively analyzing the information, thinking clearly, and rationally (Li, 2022). Several students have mastered the skills in acquiring appropriate learning strategies and studying (O’Bannon et al., 2017). One of those skills is critical thinking skills through which the students can sharpen their cognitive abilities and come up with better and more creative ideas. This generally helps the students in the long run as once the skills are acquired by the students, the students can take advantage whenever required. Moreover, critical thinking skills are best acquired in M-learning as it promotes constructive learning environments for the students (Varenina et al., 2021).

Students have an extensive spectrum of personal differences and based on this they acquire the most suitable learning which shapes their skills (Hadzhikoleva et al., 2019). One of the learning styles is the inquiry learning style. Inquiry learning is referred to as a learning process that involves the students making real-world connections by exploring and asking high-level questions (Müller and Mildenberger, 2021). This learning approach focuses on students who develop their work plan and establish real, close, and observable issues that are linked with their interests and that can be formulated with teachers’ guidance. From this approach, the school promotes the development of scientific skills and competency for solving problems using M-learning tools. A recent study conducted by Abril-López et al. (2021) showed that inquiry-based learning is one of the most effective and used methods in instruction in an M-learning environment.

Another learning style commonly found in classrooms is the reflective thinking style. In this learning style, the student takes the bigger picture and understands all of its consequences (Thiagraj et al., 2021). Reflective thinking is a significant theme in education, therefore the role of reflective thinking in M-learning that has been investigated as a crucial approach to student development (Berweger et al., 2022). Reflection is regarded as a constructive teaching practice that is narrated by the teacher when something is experienced by them. A great amount of research has been conducted in reflective thinking, and according to the researchers this learning style proves to be highly significant in the M-learning context (O’Bannon et al., 2017).

A major factor that influences the entire learning environment is peer communication. Peer communication is a form of cooperative learning that maximized the value of student-to-student interaction and brings about various benefits of learning outcomes (Wang, 2021). Hwang et al. (2018) stated that peer communication includes interactive skills and collaborative skills. Peer communication has been identified as a significant factor that shapes the learning outcomes of the students. For example, Shukla (2021) found that peer communication of students influences HOTSs in a constructive learning environment offered through M-learning. Similarly, Hwang et al. (2018) also found that interaction and collaboration have a positive association with HOTS in a mobile learning environment.

To date, few studies have been conducted from students perspectives on examining the relationships between online learning style and students’ HOTSs, when they are instructed in a mobile learning (M-learning) environment (Lu et al., 2021). Moreover, Hwang et al. (2018) stated that few studies have examined the role of online learning style in a mobile learning environment on students’ HOTS, such as problem-solving skills and critical thinking skills. To avoid pedagogical pitfalls and to develop M-learning activities for engaging the students in it, teachers and students need to understand the most important learning style to enhance students’ HOTS. Therefore, to address this gap in the literature and determine the role of the most important online learning styles, the present study aimed to examine the role of two online learning styles, that is, inquiry learning and reflective thinking on students’ problem-solving skills and critical thinking skills. Moreover, the mediating role of students’ peer communication in the learning context has not been explored yet, thus the present study aimed to examine the mediating role of students’ peer communication.

To fill this gap in literature, objectives of the study have to be addressed which are: (1) to examine the role of inquiry learning on the problem-solving skill, (2) to determine the role of inquiry learning on critical thinking skills, (3) to understand the role of reflective thinking on the problem-solving skill, (4) to analyze the role of reflective thinking on critical thinking skill, to understand the mediating role of peer communication in the relationship between inquiry learning and problem-solving skill, (5) to analyze the mediating role of peer communication in the relationship between reflective thinking and problem-solving skill, (6) to explore the mediating role of peer communication in the relationship between inquiry learning and critical thinking skill, (7) and to examine the mediating role of peer communication in the relationship between reflective thinking and critical thinking skill.

## Literature Review and Hypotheses Development

The present study intends to analyze the role of two online learning styles on two HOTSs among college students in China. For this purpose, inquiry learning and reflective thinking have been taken as online learning styles, problem-solving skills, and critical thinking skills that have been taken as HOTS. Moreover, the study also investigated the mediating role of peer communication in the relationship between inquiry learning and problem-solving skills, between inquiry learning and critical thinking skills, between reflective thinking and problem-solving, and between reflective thinking and critical thinking skills. To support the framework of the study, self-efficacy theory and social cognitive theory have been incorporated.

### Self-Efficacy Theory

Bandura and Adams (1977) developed self-efficacy theory which states that the beliefs of the people in their capabilities to have control over their functions and events influence their lives. The sense of self-efficacy in an individual provides the foundation for wellbeing, motivation, and personal accomplishment. In the light of this theory and the framework of the present study, HOTSs, that is, problem-solving skills and critical thinking skills are achieved as a result of learning styles provided in M-learning environment where the students have their support. High self-efficacy reflects confidence in students to exert control on their behavior, motivation, and environment, allowing them to become advocates of their support and needs.

### Social Cognitive Theory

The social cognitive theory developed by Bandura (2001) is commonly used in education, psychology, and education. According to this theory, the knowledge acquisition of an individual is directly associated with observing others within the context of experience, social interactions, and outside media influences. Linking this theory with the framework of the present study, this study includes peer communication which is the foundation of this theory. Based on social cognitive theory, peer communication enhances the knowledge of the individual, which has also been shown in the framework that peer communication influences the thinking skills of the students in the M-learning environment.

### Inquiry Learning Style and Problem-Solving Skills

The concept of problem-solving gets the skills needed to collect and implement the information that was found on the internet (Lin et al., 2021). Within M-learning inquiry learning students come across different kinds of problems that can be solved from information over the internet (Tan et al., 2022). The students with an inquiry learning style can develop problem-solving skills because such students engage in data collection from various sources to find the most appropriate and accurate solution to the problem (Sejati et al., 2021). Additionally, several studies have found that the problem-solving skills of the students are shaped by their learning styles. However, students attempting to self-regulate their learning use ineffective strategies and avoid help-seeking behavior (Lewis and Estis, 2020). For example, students solving a problem without thinking about the purpose behind it might face difficulty in evaluating the information sources and information results (Mauliza et al., 2022). Moreover, Farahian et al. (2021) examined how inquiry learners solve a problem through their problem-solving skills and the results showed that inquiry learners identify every possible solution to a problem and then make the right and best decision. Hwang et al. (2018) suggested investigating the role of inquiry learning on problem-solving skills that has limited literature in this regard. Therefore, to examine this relationship, the following hypotheses have been formulated:

*H1*: Inquiry learning affects problem-solving skill.

### Inquiry Learning and Critical Thinking Skills

The inquiry learning approach utilized by the students uses a chemical learning method that focuses on reaction rate material and is student-centric (Ojiako et al., 2011). Inquiry learning style is a learning model adopted by the students to discover concepts, principles, phenomena, or events by experiencing from a lesson as the teacher only facilitates and supports (Faradita et al., 2022). This learning style involves questions and ideas that can stimulate the students to develop different ways to communicate what they have already learned. Critical thinking is logical and reflective thinking that focuses on what to believe and what actions to take (Gencel et al., 2021). Additionally, critical thinking helps in solving the problem and improving knowledge. The students who possess this learning style are actively engaged in investigating their knowledge, ensuring self-sufficiency, engagement, and capability of solving challenges based on awareness and knowledge (Kamal and Radhakrishnan, 2019). Significantly, an inquiry is a learning approach developed to educate students on thinking critically, thus developing their critical thinking skills (Isaac et al., 2019). Additionally, the inquiry learning style fosters critical thinking skills by collecting data and information from various sources. Based on this understanding, Müller et al. (2021) investigated the impact of inquiry learning style on critical thinking among Physics students in an M-learning environment and found that these variables are positively related. However, the investigation of this relationship is yet to be explored (Lu et al., 2021), thus, based on past studies and to fill the gap in the literature, the following hypotheses have been developed:

*H2*: Inquiry learning affects critical thinking skills.

### Reflective Thinking and Problem-Solving Skills

The reflective thinking style adopted by the individuals allows them to take a bigger picture and understand all the consequences. Based on this, the students conduct extensive research which ultimately helps in problem-solving (Kholid et al., 2020). A recent study conducted by MacLeod et al. (2018) examined the relationship between reflective thinking style toward problem-solving in achieving science and technology courses in a virtual environment. The result of the study showed that reflective thinking style is a precursor of problem-solving skills in achieving science and technology course. Similarly, Toker and Akbay (2022) studied the impact of reflective thinking style on problem-solving skills and attitudes of students studying in China. Findings showed that reflective thinking skills shape and enhance problem-solving skills and attitudes of the students as the student indulges in information collection which sharpens his problem-solving abilities. Moreover, Tanujaya et al. (2017) worked on the influence of learning styles on HOTS among college students and found that the problem-solving abilities of the students are influenced by HOTS. Lu et al. (2021) suggested further investigation on the relationship between reflective thinking style and problem-solving skills in an M-learning environment. Based on past studies and to fill the gap in the literature, the following hypotheses have been formulated:

*H3*: Reflective thinking affects problem-solving skill.

### Reflective Thinking and Critical Thinking Skills

Cooperative learning requires both reflective thinking and critical thinking skills (Calma and Davies, 2020). Critical thinking is logical and reflective thinking that focuses on what to believe and what actions to take. Additionally, critical thinking helps in solving the problem and improving knowledge. Critical thinking related to English skills, such as questioning, problem-solving, and analyzing, is an integral part of English education (Kholid et al., 2020). The nature of critical thinking compels one to acquire sociability and reflection (Toker and Akbay, 2022). According to MacLeod et al. (2018), reflective thinking requires critical thinking due for this reason reflective thinking is associated with critical thinking skills. Moreover, a reflective thinking student has high critical thinking abilities. Studies show that activities of students to improve their reflective thinking learning style have the potential to influence critical thinking skills (Cortázar et al., 2021). Considering research carried out in China, especially on the learning environment, few studies have been found to investigate the role of reflective thinking on critical thinking skills (Lu et al., 2021). Based on the literature reviewed above, the following hypotheses have been established:

*H4*: Reflective thinking affects critical thinking skill.

### Peer Communication as a Mediator

Peer communication is a form of cooperative learning that increases the interaction among students and results in various benefits of learning outcomes (Shukla, 2021). Peer communication involves communication skills that help the students to have a conversation with each other and collaboration which enables different ideas from every student (Hwang et al., 2018). Specifically, collaboration is the individual’s ability to work together in groups and share their ideas and thoughts to achieve the learning goals and accomplish learning tasks. Communication, on the other hand, refers to the ability of an individual to circulate ideas and perceptions effectively with the use of oral, non-verbal, and written communication skills in different contexts and ways (Zook and Ryall, 2021). Past literature showed that peer communication significantly impacts the learning outcomes of students. For example, Tanujaya et al. (2017) studied that peer communication of students could be a key predictor of HOTSs in a constructive learning environment. Moreover, collaboration and communication have a positive and significant impact on HOTS in the learning environment (Hwang et al., 2018).

Additionally, existing research shows that both learning styles, that is, inquiry learning and reflective thinking have a positive association with peer communication (Ala et al., 2021). For instance, Ismailov (2021) found that inquiry learning had a positive relationship with the peer communication of students in an interactive blogging learning environment. Apart from this, Wulandari (2022) found that reflective thinking and peer communication was positively related in the context of a computer-supported collaborative environment. Moreover, Wilczewski et al. (2021) investigated the impact of online learning styles on peer communication among students in a collaborative environment. The results revealed that the online learning style of the students directly impacts peer communication among them such that the students who possess inquiry-based learning would indulge in peer communication because they need new information and peers are the sources of information. Similarly, students with a reflective thinking style take a bigger picture and understand all the consequences, and this bigger picture can be acquired by communicating with other peers and taking their thoughts and perceptions (Gencel et al., 2021).

The literature showed the examination of inquiry-based learning as an instructional approach to investigate the role of inquiry learning and peer interaction on students’ HOTS. Several universities have used collaborative inquiry learning styles to develop collaborative, critical, and problem-solving skills (MacLeod et al., 2018). However, previous studies have found classroom interaction has a positive impact on HOTS (Sinha and Kapur, 2021). But few studies have examined peer communication as a mediator in the relationship between online learning skills and HOTS in a collaborative M-learning context (Lu et al., 2021).

**Figure 1:**
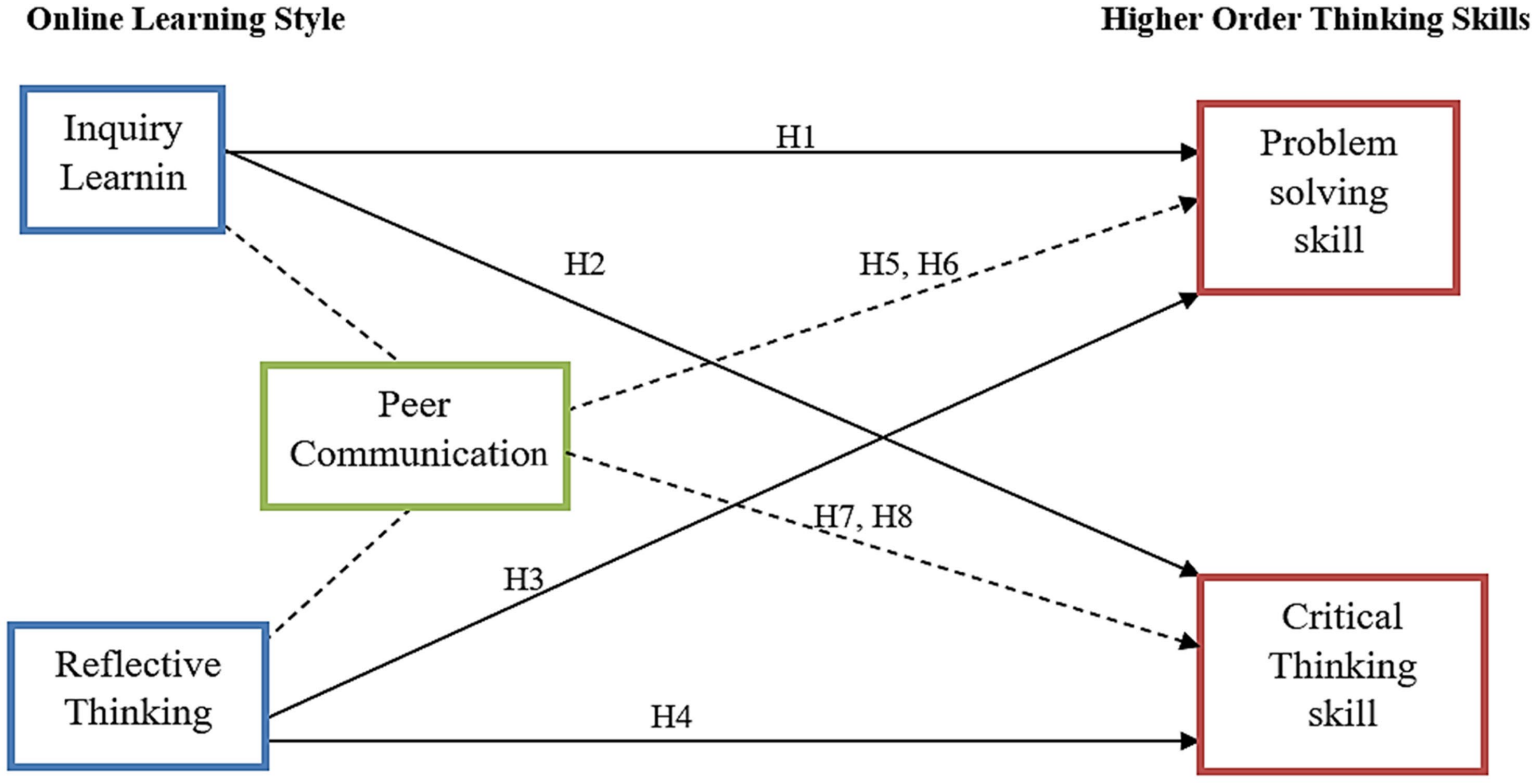
Theoretical framework.

Peer communication has been studied in the context of buying behavior, for example, a study conducted by Harrigan et al. (2021) examined the role of consumer buying behavior influenced by peer communication, and the result showed that peer communication is a significant tool in fostering individual’s behavior. Another study was carried out by Müller et al. (2021) to investigate the impact of peer communication among students in achieving higher aggregate and the result revealed that peer communication immensely helped the students to achieve higher grades. Similarly, Ala et al. (2021) also studied the influence of peer interaction among university students in China and found that peer interaction enables students to gain experience, learn more, and broaden their horizons. Although studies have been conducted on peer communication, limited studies have examined peer communication as a mediator in the relationship between online learning skills and HOTS in a collaborative M-learning context (Lu et al., 2021). Therefore, to examine this relationship, the following hypotheses have been formulated (Figure 1):

*H5*: Peer communication mediates the relationship of inquiry learning and problem-solving skill.

*H6*: Peer communication mediates the relationship of reflective thinking and problem-solving skill.

*H7*: Peer communication mediates the relationship of inquiry learning and critical thinking skill.

*H8*: Peer communication mediates the relationship of reflective thinking and critical thinking skill.

## Methodology

This section entails the methodology that was adopted to examine and investigate the impact of inquiry learning, reflective thinking on problem-solving and critical thinking skills through the mediating mechanism of peer communication. A research methodology holds utmost significance because it aids the researcher in laying out a clear and comprehensive methodological plan for particular research. Moreover, a clear methodological plan is also important because it adds to the overall integrity and credibility of the study (Marchena-Giráldez et al., 2021). The present study adopted the research onion technique to formulate a research methodology. A research onion is a step-wise approach to creating a methodological plan. It includes the overall design of research, population and sample size, sampling procedure, unit of analysis, time horizon, instrumentation, data collection, and data analysis techniques.

### Research Design

A research design is usually referred to as the overall approach that is adopted by a researcher to examine and investigate a particular phenomenon. A research design lays the foundation of the methodological plan of a particular study (Nawaz et al., 2022). Therefore, it becomes imperative to select an appropriate research design to accomplish the research objectives effectively. Currently, many research designs are being used by researchers around the world. The positivist approach posits that the reality of any particular phenomenon can truly be examined without any sort of external human interference (Avotra et al., 2021; Dar et al., 2022). This design further advocates the use of quantitative techniques for research. These quantitative techniques include surveys, paper and pencil tests, and other statistical and computational methods (Sarstedt and Cheah, 2019). The current study adopted a positivist research design by deploying various quantitative techniques to investigate the topic under study. A positivist approach was used because it involves the use of quantitative techniques and the data is backed by strong facts, figures, and empirical evidence. Data that is backed by strong numerical facts are more effective, dependable, and credible. Moreover, the primary nature of this research perfectly aligns with the adoption of positivist research design.

### Population

A population or universe is commonly referred to as the main area of focus and interest of the study. The target population varies from one to another study depending upon the topic and the context of the research. A population may be comprised of an individual(s), group(s), community, etc. The identification of the right target population is necessary to generate credible and accurate results and findings (An et al., 2021; Xiaolong et al., 2021). The target population of this study was comprised of students who are currently studying in various colleges in China. The colleges were chosen because the review of the literature indicates high school and college students that are more involved in the use of technological gadgets and tools during their studies. These tools and gadgets aid the students to enhance the overall learning process. Moreover, certain demographic characteristics of the population were taken into account during this study. These demographic characteristics included gender, age, education, and field of study.

### Sample Size

A sample is defined as a subset of the population that serves as a representative of the target population since it is impossible to study the entire population (Adam, 2020). Therefore, a researcher needs to determine a suitable and appropriate sample size to improve the generalizability of a study. Various techniques are being used by researchers to determine suitable sample sizes and there does not seem to be a consensus upon the accuracy of any particular technique for determining sample size (Stratton, 2021). Roscoe’s rule of thumb developed by Roscoe et al. (2018) is a widely used technique to determine a suitable sample size. According to this rule of thumb, a sample size of more than 30 and less than 500 participants is sufficient and appropriate for conducting particular research. On the other hand, Tanaka (1987) proposed the item response theory to determine sample size. According to the item response theory, at least 10 responses are required against each item of the measurement scale. By applying the item response theory, a researcher can determine the sample size by simply multiplying the total number of items in the measurement scale by 10. Both of the aforementioned techniques that are widely used and significantly aid the researcher in determining a suitable sample size effectively and efficiently.

The present study adopted Roscoe’s rule of thumb to determine a suitable sample size. By applying this technique, the sample size has been determined to be 450 respondents. This technique was used because it is more cost-effective and aids in determining sample size in an effective and timely manner.

### Sampling Procedure

Once the sample size has been determined, it becomes necessary to adopt a suitable sampling technique to acquire data from the participants of the study. The sampling techniques are classified into two major categories, namely probability and non-probability sampling techniques. In a probability sampling technique, each member of the target population has an equal chance of selection whereas in a non-probability sampling technique each member does not have an equal chance of being selected. The probability sampling techniques include cluster sampling, quota sampling, strata sampling, etc. The non-probability sampling techniques include random sampling, convenience sampling, snowball sampling, purposive sampling, etc. (Nawaz et al., 2019).

The present study adopted a non-probability convenience sampling technique to obtain data from the participants. In a convenience sampling technique, the data is acquired from the most readily and conveniently available respondents. This technique was adopted because it facilitates acquiring data within a short time and cost-effectively and efficiently (Etikan et al., 2015).

### Unit of Analysis

A unit of analysis usually refers to the main entity that is being analyzed in a particular study. A unit of analysis can be individual or it may involve group(s) or community etc. The unit of analysis for this study was individual because it was focused on the individual students who are currently studying at various colleges in China.

### Time Horizon

The time horizon can be described as the time frame within which the research is expected to be completed. The time horizon can be classified into two major categories, namely, longitudinal and cross-sectional time horizon (Dash and Paul, 2021). In a longitudinal time, horizon, the data are obtained from the respondents at multiple points of time whereas, in a cross-sectional time horizon, the data is acquired at a single point of time. The time frame of this study was cross-sectional because the data was acquired at a singular point in time.

### Measurement

The measurement scales for this study were adopted from renowned databases and studies that have been undertaken in a similar context. The measurement scales were classified into two sections. The first section comprised of the demographic information of the respondents, that is, gender, age, and education. The second section consisted of the questions aimed at measuring the constructs of this study. Moreover, a five-point Likert scale (i.e., 1 = strongly disagree and 5 = strongly agree) was adopted to gauge the responses from the participants of the study. The measurement scale consisted of a clear introduction indicating the purpose of the study along with instructions for filling the questionnaire. The variables along with the items of their measurement scales and the sources from where they have been adopted are given below.

#### Inquiry Learning

The scale of inquiry learning consisted of five items, and it was adopted from MacLeod et al. (2018).

#### Reflective Thinking

There were five items in the scale of reflective thinking, and it was adopted from MacLeod et al. (2018).

#### Peer Communication

The Peer communication scale had six items, and it was adopted from Hwang et al. (2018).

#### Problem-Solving Skills

The scale of problem-solving skills had four items, and it was adopted from Hwang et al. (2018).

#### Critical Thinking Skill

There were four items on the scale of critical thinking skills, and it was adopted from Hwang et al. (2018).

### Data Collection

The data was obtained from the respondents through the aid of a self-administered survey. The survey form consisted of clear information indicating the underlying purpose of the study along with detailed instructions for the respondents to fill out and return the survey forms (Ferreira et al., 2016). Sufficient time was allocated to the respondents to return the survey forms and all queries were addressed effectively. The respondents were told to be as natural as possible when filling out the survey form to generate credible results. A total of 450 survey forms were disbursed out of which 378 were considered to be appropriate for this study. The researcher took 3 weeks to obtain and screen the data. During the process of screening, 72 responses were discarded and the overall response rate was 84%.

### Statistical Tool

Once the data was obtained, it was arranged and examined through the use of Smart-PLS 3.3.3 software. The Smart-PLS facilitates in providing deep insights on the data through the creation of path models within a short time (Hair et al., 2011). Moreover, a structural equation modeling (SEM) technique was applied to examine the data. Under this technique, the measurement and structural models were assessed. The measurement model provides insights on indicators, such as data reliability, AVE, factor loadings, VIF values, HTMT ratios, and Fornell and Larcker criterion. On the other hand, the structural model is used to determine the validities of the proposed hypotheses through the aid of values of *p*, *f*-values, and *t*-statistics.

### Demographic Profile

The analysis of the demographic characteristics of the respondents indicates that there were 215 males and 163 females who participated in this study. The males and females constituted 56.8 and 43.1% of the sample size, respectively. Moreover, it can be seen that 252 respondents were aged between 20 and 30 years whereas, 126 respondents were aged between 31 and 40 years. It can also be observed that 201 respondents possessed a Bachelor’s education, 115 respondents had a Master’s degree whereas 62 respondents had an M Phil qualification as depicted in Table 1. As far as the field of study is concerned it can be observed that 157 respondents were students of management sciences, 162 students belonged to the discipline of social sciences, whereas 59 students had an engineering background.

**Table 1:** Demographics analysis.

Demographics	Frequency	Percentage (%)
**Gender**		
Male	215	56.87
Female	163	43.13
**Age (years)**		
20–30	252	66.66
31–40	126	33.34
**Education**		
Bachelors	201	53.17
Masters	115	30.43
M. Phil.	62	16.40
**Field of study**		
Management Sciences	157	41.53
Social Sciences	162	42.86
Engineering	59	15.61

*N* = 378.

## Data Analysis and Results

### Measurement Model

**Figure 2:**
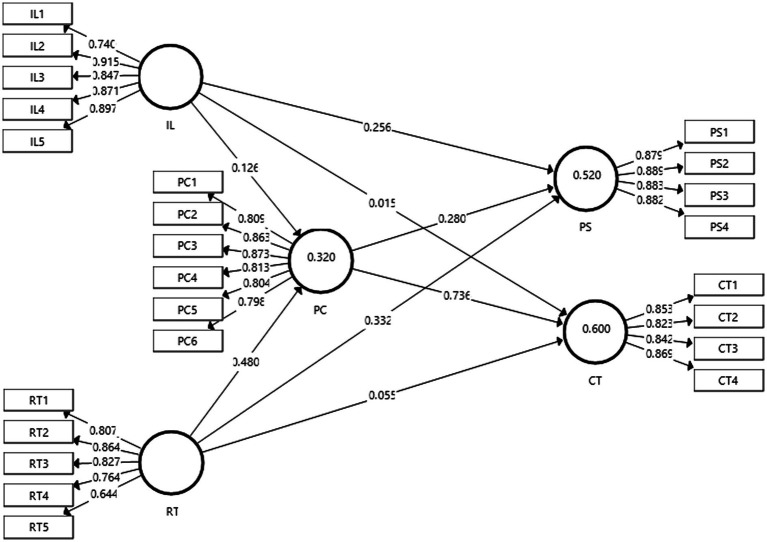
Output of measurement model. IL, inquiry learning; RT, reflective thinking; PS, problem-solving skills; CT, critical thinking skills; and PC, Peer Communication.

The figure shown below, that is, Figure 2 depicts the outcome of the measurement model. The figure shows the extent or degree to which the predictor constructs affect the outcome variables that are being studied.

Table 2 below demonstrates the factors loadings of the constructs along with the VIF values that were obtained for each item of inquiry learning, reflective thinking, peer communication, problem-solving skills, and critical thinking skills. According to Jordan and Spiess (2019), the acceptable factor loadings should be greater than 0.60. It can be observed that all factor loadings ranged between 0.644 and 0.915. Therefore, it can be concluded that all factor loadings were satisfactory. In addition to this, the variance inflation factor (VIF) values were also obtained. The VIF is a measure that indicates collinearity within the proposed model. As per Henseler et al. (2015) all VIF values should be less than 5. It can be observed from the table that all VIF values satisfied this assumption. Hence, it can be ascertained that the present study had no issues of collinearity.

**Table 2:** Model assessment (direct model).

	Factor loadings		VIF	Construct reliability and validity		
				*α*	Composite reliability	AVE
**Inquiry learning**	IL1	0.740	1.467	0.908	0.932	0.733
	IL2	0.915	4.285			
	IL3	0.847	2.705			
	IL4	0.871	3.499			
	IL5	0.897	4.073			
**Reflective thinking**	RT1	0.807	1.926	0.842	0.888	0.616
	RT2	0.864	2.801			
	RT3	0.827	2.316			
	RT4	0.764	1.669			
	RT5	0.644	1.415			
**Problem-solving skills**	PS1	0.879	2.558	0.906	0.934	0.780
	PS2	0.889	2.783			
	PS3	0.883	2.579			
	PS4	0.882	2.646			
**Critical thinking skills**	CT1	0.853	2.203	0.869	0.910	0.717
	CT2	0.823	2.095			
	CT3	0.842	2.017			
	CT4	0.869	2.225			
**Peer communication**	PC1	0.809	2.752	0.908	0.928	0.684
	PC2	0.863	3.090			
	PC3	0.873	3.743			
	PC4	0.813	2.530			
	PC5	0.804	2.314			
	PC6	0.798	2.474			

IL, inquiry learning; RT, reflective thinking; PS, problem-solving skills; CT, critical thinking skills; PC, Peer communication; VIF, variance inflation factor; α, Cronbach alpha; and AVE, average variance extracted.

In addition to this, the values of construct reliabilities and validities are also given in Table 2. The reliabilities and validities were assessed using the indicators of Cronbach’s alpha (*α*), composite reliability (CR), and average variance extracted (AVE). Hair et al. (2011) posited that a particular construct is reliable if its Cronbach’s alpha value is greater than 0.70. Furthermore, according to Peterson and Kim (2013), the acceptable values of composite reliability should be higher than 0.70. It can be observed that all values of Cronbach’s alpha and CR successfully met the aforementioned assumptions. Moreover, Diputra et al. (2021) stated that the desirable values of AVE should be higher than 0.60. This assumption was also successfully met as all values of AVE were above 0.60. Based on these tests, it can be deduced that convergent validity was present in the proposed model.

To determine the presence of discriminant validity, the heterotrait–monotrait ratio (HTMT) and the Fornell and Larcker criterion was adopted. The results of these tests can be seen in Table 3 which is given below. Discriminant validity is a measure that indicates whether or not one particular construct is different from the other. It can be seen that all HTMT values are below that acceptable threshold of 0.90 which suggests that discriminant validity was present within the model (Benitez et al., 2020). As far as the Fornell and Larcker criterion is concerned, the general assumption is that all values at the top column must be higher than the values that lie below them (Fornell and Larcker, 1981). This assumption was also successfully met. Thus, it can be concluded that discriminant validity was present in between the constructs of the study.

**Table 3:** Discriminant validity.

Fornell–Larcker criterion					Heterotrait–Monotrait ratio				
Constructs	CT	IL	PC	PS	RT	Constructs	CT	IL	PC	PS	RT
**CT**	0.847					**CT**					
**IL**	0.355	0.856				**IL**	0.387				
**PC**	0.773	0.417	0.827			**PC**	0.863	0.440			
**PS**	0.588	0.574	0.572	0.883		**PS**	0.665	0.620	0.622		
**RT**	0.474	0.605	0.557	0.643	0.785	**RT**	0.546	0.682	0.626	0.731	

*N* = 378; IL, inquiry learning; RT, reflective thinking; PS, problem-solving skills; CT, critical thinking skills; and PC, Peer communication.

The R and Q-square values obtained against critical thinking skills, peer communication, and problem-solving skills are shown in Table 4 which is given below. Franke and Sarstedt (2019) stated that the R-square is a denotation of the overall sustainability of the model and that the desirable values of R-square should lie in proximity to 0.50. It can be observed that the R-square values obtained against critical thinking skills, peer communication, and problem-solving skills were 0.600, 0.320, and 0.520, respectively. This depicts that the proposed model is sustainable. The values of Q-square are a denotation of cross-validated redundancy. Hair et al. (2011) posited that the desirable values of Q-square should be higher than zero. It can be observed that all values of Q-square successfully met this assumption. Therefore, it can be ascertained that the proposed model is fit and sustainable.

**Table 4:** R-Square values and Q-Square values for the variables.

	R-Square	Q-Square
**CT**	0.600	0.146
**PC**	0.320	0.190
**PS**	0.520	0.321

IL, inquiry learning; RT, reflective thinking; PS, problem-solving skills; CT, critical thinking skills; and PC, Peer communication.

The inner VIF values depicting collinearity within the proposed model are depicted in Table 5. According to Hair et al. (2011), the inner VIF values should be lower than 5. This assumption was successfully met as all inner VIF values were below 5. Hence, it can be deduced that collinearity did not exist between the constructs of this study.

**Table 5:** Collinearity statistics (inner VIF values).

	CT	IL	PC	PS	RT
**CT**					
**IL**	1.602		1.579	1.602	
**PC**	1.471			1.471	
**PS**					
**RT**	1.918		1.579	1.918	

IL, inquiry learning; RT, reflective thinking; PS, problem-solving skills; CT, critical thinking skills; and PC, Peer communication.

### Structural Model

**Figure 3:**
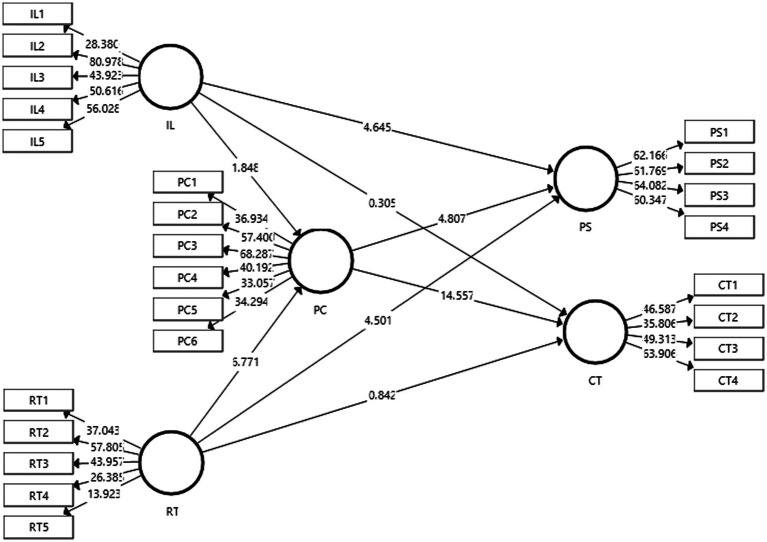
Structural model Bootstrapping. IL, inquiry learning; RT, reflective thinking; PS, problem-solving skills; CT, critical thinking skills; and PC, Peer Communication.

The outcome of the structural model bootstrapping is shown in Figure 3. It also denotes the values of *t*-statistics. The validities of the proposed hypotheses were assessed through the aid of the PLS-SEM bootstrapping model that was undertaken at confidence intervals of 95%.

Tables 6 and 7 depict the outcome of the direct and indirect effects that were observed to assess the validities of the proposed hypotheses. The *t*-statistic and value of *p* denote the acceptance or rejection of a certain hypotheses. According to Johnson (2019), the desirable *t*-value should be greater than 1.96. Moreover, Di Leo and Sardanelli (2020) stated that the desirable values of *p* should be less than 0.05. The effect sizes are also denoted through the *f*^2^ which depicts the strength/weakness of the proposed model. Meng and Bari (2019) posited that the strength of the model is highest when the *f*^2^ is close to 1 and the model strength is weak if it is close to 0.

**Table 6:** Direct effects of the variable.

Paths	H	O	M	SD	T-statistics	Effect size (*f*^2^)	*p*	Results
IL → PS	H_1_	0.256	0.253	0.055	4.645	0.085	0.000***	***Accepted***
IL → CT	H_2_	0.015	0.011	0.048	0.305	0.000	0.761	*Rejected*
RT → PS	H_3_	0.332	0.339	0.074	4.501	0.120	0.000***	***Accepted***
RT → CT	H_4_	0.055	0.059	0.065	0.842	0.004	0.4000	*Rejected*

H, hypotheses; O, original sample; M, sample mean; SD, standard deviation; SRMR = 0.082; NFI = 0.793; IL, inquiry learning; RT, reflective thinking; PS, problem-solving skills; CT, critical thinking skills; and PC, Peer communication.

**Table 7:** Indirect effects of the variable.

Paths	H	O	M	SD	*t*-statistics	*p*	Results
IL → PC → PS	H_5_	0.035	0.035	0.022	1.595	0.111	*Rejected*
RT → PC → PS	H_6_	0.134	0.132	0.030	4.515	0.000	***Accepted***
IL → PC → CT	H_7_	0.093	0.090	0.052	1.793	0.074	*Rejected*
RT → PC → CT	H_8_	0.353	0.355	0.056	6.285	0.000	***Accepted***

H, hypotheses; O, original sample; M, sample mean; SD, standard deviation; IL, inquiry learning; RT, reflective thinking; PS, problem-solving skills; CT, critical thinking skills; and PC, Peer communication.

Table 6 depicts the results of the proposed hypotheses; H1, H2, H3, and H4. Hypotheses (H1) proposed that inquiry learning (IL) had an effect on problem-solving skills (PS). The t-statistic and values of *p* are (*t* = 4.645, *p* = 0.000) which indicates the acceptance of these hypotheses. The *f*^2^ value is 0.085 which suggests that the model strength is moderate to strong. H2 predicted that inquiry learning (IL) had an effect on critical thinking skills (CT). The corresponding t-statistic and values of *p* are (*t* = 0.305, *p* = 0.761) which indicates that these hypotheses have been rejected. H3 proposed that reflective thinking (RT) had an effect on problem-solving skills (PS). The values of *t* and *p* are (*t* = 4.501, *p* = 0.000) which indicates that reflective thinking has an effect on problem-solving skills. Therefore, these hypotheses have been accepted. The effect size is 0.120 which means that model strength is low. H4 predicted that reflective thinking (RT) had an effect on critical thinking skills (CT). The corresponding values of *t* and *p* are (*t* = 0.842, *p* = 0.4000) which indicates that these hypotheses have been rejected.

The fitness of the proposed model was assessed using the normed fixed index (NFI) criterion. The desirable value of NFI should lie between 0 and 1 (Hair et al., 2011). The value of NFI was 0.793 which indicates higher model fitness. Moreover, Meng and Bari (2019) stated that the desired value of standardized root mean square residual (SRMR) should be between 0 and 0.09. The value of SRMR was recorded at 0.082. Therefore, it can be ascertained that the proposed model was fit for the data.

Table 7 shows the outcome of the indirect effects of the constructs. The fifth hypotheses, H5 proposed that peer communication (PC) mediated between inquiry learning (IL) and problem-solving skills (PS). The values of *t* and *p* were 1.595 and 0.111, respectively. These values indicate that these hypotheses have been rejected. H6 proposed that peer communication (PC) mediated between reflective thinking (RT) and problem-solving skills (PS). The corresponding values of *t* and *p* were 4.515 and 0.000, respectively, which means that these hypotheses have been accepted. H7 predicted that peer communication (PC) mediated between inquiry learning (IL) and critical thinking skills (CT). The values of *t* and *p* of 1.793 and 0.074 indicate that these hypotheses have been rejected. Lastly, H8 predicted that peer communication (PC) mediated between reflective thinking (RT) and critical thinking skills (CT). The values of t-statistic and *p* were 6.285 and 0.000 which suggest that these hypotheses have been accepted. Therefore, it can be concluded that peer communication mediated the relationship between reflective thinking and problem-solving skills and, reflective thinking and critical thinking skills.

## Discussion

The data was collected from college students studying in China to fill the gap in the literature about mobile learning. Generally, the study investigated the role of online learning styles on HOTS in an M-learning environment. Initially, the study examined the direct relationships, that is, the impact of inquiry learning on problem-solving skills and critical thinking skills. The study also investigated the role of other learning styles, that is, reflective thinking on problem-solving skills and critical thinking skills. Additionally, the present study also included the mediating role of peer communication in the relationship between inquiry learning and problem-solving skills and critical thinking skills. Also, the mediating role of peer communication was determined in the relationship between reflective thinking and problem-solving skills, and critical thinking skills. Some useful insights regarding learning styles and HOTS in M-learning environments were provided from the results obtained. The study helped to provide key recommendations for colleges.

The first hypotheses of the present study was accepted meaning inquiry learning has an effect on problem-solving skills among college students in the context of M-learning. The findings of this study were in harmony with the study carried out by Sejati et al. (2021) which explained that the students who possess this learning style are actively engaged in investigating their knowledge, ensuring self-sufficiency, engagement, and capability of solving challenges based on awareness and knowledge. The reason could be the inquisitive nature of the students that prompts them to unearth the problem and take out the best possible solution. The second hypotheses of the present study was rejected meaning inquiry learning has no effect on problem-solving skills among college students in the context of m-learning. This result contradicted the findings of Farahian et al. (2021) who found that reflective thinking style is a precursor of problem-solving skills in achieving Science and Technology course. The possible reason could be reflective thinking focuses on a broader picture and understanding the consequences rather than finding the solution of a problem.

The third hypotheses of the present study was accepted meaning reflective thinking has an effect on problem-solving skills among college students in the context of M-learning. Similar results were acquired by Toker and Akbay (2022), which explained that reflective thinking skills shape and enhance problem-solving skills and attitudes of the students as the student indulges in information collection which sharpens his problem-solving abilities. This could be because reflective thinkers engage themselves in findings the best possible solution to a problem and during this process, they build and develop problem-solving abilities. The fourth hypotheses of the present study was rejected meaning reflective thinking has an effect on critical thinking skills among college students in the context of m-learning. However, Cortázar et al. (2021) found that activities of students to improve their reflective thinking learning style have a positive influence on critical thinking skills. The fourth hypotheses of the study might get rejected because a reflective thinker aims to find the outcomes of something which can develop his problem-solving abilities but not cognitive or critical thinking skills.

The fifth and seventh hypotheses of the study were rejected suggesting that peer communication did not mediate the relationship between inquiry learning and problem-solving skills nor in the relationship between inquiry learning and critical thinking skills. These results contradicted the findings obtained by Tanujaya et al. (2017) who stated that peer communication of students could be a key predictor of HOTSs in a constructive learning environment. The reason could be that inquiry learners do not rely on peer interaction while finding a solution to something, especially in an M-learning environment where mobile devices can be used to obtain a wide range of information available over the internet. These learners find internet sources to be more reliable and accurate than peers.

The sixth and eighth hypotheses of the study were accepted suggesting that peer communication mediated the relationship between reflective thinking and problem-solving skills and in the relationship between reflective thinking and critical thinking skills. Similarly, Wulandari (2022) also found that reflective thinking and peer communication was positively related in the context of a computer-supported collaborative environment. The plausible reason could be that reflective thinkers focus on a broader picture and understand the consequences, therefore to take a bigger picture they take information from peers and the internet to understand all the consequences.

## Theoretical and Practical Implications, Limitations and Future Directions, and Conclusion

### Theoretical Implications

The present study greatly contributes to the current literature by investigating the role of online learning styles, that is, inquiry learning and reflective thinking on HOTSs, that is, problem-solving skills and critical thinking skills. As fewer studies were conducted in this regard, especially in the mobile learning environment, the present study greatly advances the present literature. These results provided insights into this phenomenon by finding that reflective thinking is positively related to both problem-solving skills and critical thinking skills in M-learning environment, therefore, reflective thinking must be optimized in M-learning environment. Also, the results showed that inquiry learning is not related to both problem-solving skills and critical thinking skills in an M-learning environment, thus such skills are increased using other learning styles rather than inquiry learning. This study provided another valuable contribution by showing that peer communication helps to enhance HOTS with reflective thinking style rather than inquiry learning style in an M-learning environment.

### Practical Implications

The present study also possesses some practical implications. The colleges providing an M-learning environment can enhance problem-solving skills and critical thinking skills by encouraging a reflective thinking learning style. This can be promoted by encouraging students to figure out all the consequences from various sources, and this process will maximize their HOTS. Moreover, the colleges must devise policies and educational programs to integrate M-learning environment to shape the students’ HOTS. Additionally, peer communication among the students must also be encouraged by the teachers by increasing the overall grade for students who are highly engaged in peer communication. This will not only improve the communication skills of reflective thinkers but also enhance their problem-solving and critical thinking skills. Furthermore, inquiry learners must be provided with problem-seeking tasks so that they can use their abilities to magnify problem-solving skills.

### Limitations and Directions for Future Studies

Like other studies, the present study also includes a few limitations that must be addressed in the future. First, more data from the study respondents must be acquired in future studies to reduce the issue of generalizability. The present study was conducted in China, therefore future studies can replicate the research framework in other cultural contexts. Third, future studies can use other learning styles, such as visual learning or verbal learning, and other HOTS, such as creativity, to examine the relationship among them. The present study did not use any moderator the study; therefore, future studies can incorporate moderators, such as teachers’ support in the present framework. Moreover, a longitudinal approach replacing a cross-sectional study design can be used in future studies.

### Conclusion

The growth of technology has been changing the learning environment and compelling the students to be more flexible, social, and interactive. Due to this reason, the concept of mobile learning emerged in which students can use their portable devices to access the mobile network to carry out their learning which can be either in or out of the classroom. There are different learning styles possessed by the students which significantly impact their HOTSs in M-learning environment. In the light of this knowledge, the current study analyzed the impact of inquiry learning on problem-solving skills and critical thinking skills. The study also examined the influence of reflective thinking on problem-solving skills and critical thinking skills. Additionally, the mediating role of peer communication was also examined in the presents study. For this reason, the data was acquired from college students studying in China. The results revealed that inclusive learning and reflective thinking affected problem-solving skills. However, inquiry learning and reflective thinking did not affect critical thinking skills. Moreover, the study found that peer communication mediated the relationship between reflective thinking and problem-solving skills and between reflective thinking and critical thinking skills. However, peer communication did not mediate the relationship between inquiry learning and problem-solving skills and between inquiry learning and critical thinking skills.

## Data Availability Statement

The original contributions presented in the study are included in the article/supplementary material, further inquiries can be directed to the corresponding author.

## Author Contributions

XC: conceptualization, data collection, writing the draft, and agreed to the submitted version of manuscript.

## Conflict of Interest

The author declares that the research was conducted in the absence of any commercial or financial relationships that could be construed as a potential conflict of interest.

## Publisher’s Note

All claims expressed in this article are solely those of the authors and do not necessarily represent those of their affiliated organizations, or those of the publisher, the editors and the reviewers. Any product that may be evaluated in this article, or claim that may be made by its manufacturer, is not guaranteed or endorsed by the publisher.
